# Expanding the spectrum of chronic hepatitis E in kidney transplantation: first report of HEV-3ra infection and review of literature

**DOI:** 10.3389/fmed.2025.1705331

**Published:** 2025-12-08

**Authors:** Emanuel F. Matias, Mariana Azevedo, Susana Sampaio, Ana Teresa Pinho, Silvia Conde, Sandra Rebelo, Sérgio Santos-Silva, Maria S. J. Nascimento, João R. Mesquita, Lurdes Santos

**Affiliations:** 1 Department of Infectious Diseases, São João University Hospital Centre, Porto, Portugal; 2 Unit of Pharmacology and Therapeutics, Department of Biomedicine, Faculty of Medicine, University of Porto, Porto, Portugal; 3 Department of Nephrology, São João University Hospital Centre, Porto, Portugal; 4 Department of Clinical Pathology, São João University Hospital Centre, Porto, Portugal; 5 Institute for Research and Innovation in Health (i3S), Porto, Portugal; 6 Laboratory for Integrative and Translational Research in Population Health (ITR), Porto, Portugal; 7 School of Medicine and Biomedical Sciences (ICBAS) of the University of Porto, Porto, Portugal; 8 Faculty of Pharmacy of the University of Porto (FFUP), Porto, Portugal; 9 Epidemiology Research Unit (EPIUnit), Institute of Public Health of the University of Porto, Porto, Portugal; 10 ESCMID Study Group for Infections in Compromised Hosts, European Society of Clinical Microbiology and Infectious Diseases, Basel, Switzerland

**Keywords:** HEV, chronic hepatitis E, immunosuppression, kidney transplant recipient, ribavirin

## Abstract

Chronic hepatitis E virus (HEV) infection is increasingly recognized as a significant cause of post-transplant hepatitis in immunosuppressed patients. Although HEV genotype 3 is the most prevalent in Europe, the clinical significance of subgenotypic diversity remains poorly defined. We report two kidney transplant recipients (KTR) diagnosed with chronic HEV infection during routine follow-up. Both patients presented with abnormal liver function tests in the absence of overt clinical symptoms. A broad-spectrum nested RT-PCR assay targeting the RNA-dependent RNA polymerase (RdRp) gene of the ORF1 region of the HEV genome was performed, followed by sequencing and phylogenetic analysis. Viral sequences detected in stool samples clustered with HEV-3c and HEV-3ra subtypes, the latter being a zoonotic variant associated with rabbits. Immunosuppressive reduction alone was insufficient for viral clearance, and both patients achieved sustained virologic response after six-month ribavirin therapy. These findings are consistent with established features of chronic HEV infection in KTR but also extend current knowledge by documenting the first case of HEV-3ra in this population. The present work underscores the importance of systematic HEV surveillance and routine molecular characterization in transplant medicine, reinforcing the need for timely initiation of antiviral therapy to prevent progression to advanced forms of liver disease.

## Introduction

Hepatitis E virus (HEV) is an emerging cause of viral hepatitis globally (1, 2). Phylogenetic analysis have identified several genotypes, HEV-1 to HEV-8, of which HEV-1, HEV-2, HEV-3, HEV-4, and HEV-7 are known to infect humans (3). While HEV-1 and HEV-2 have been found only in humans and are associated with waterborne outbreaks in developing countries, HEV-3 and HEV-4 have a broader host range and cause zoonotic infections in humans mainly through the consumption of raw or undercooked pork meat or contact with infected swine (4). While most HEV-3 and HEV-4 infections remain asymptomatic or self-limiting, immunocompromised patients are at risk of chronic liver disease that may progress rapidly to cirrhosis and liver failure (5, 6). HEV-3 represents the leading cause of chronic infection in immunosuppressed patients, in particular solid organ transplant recipients (7, 8). Kidney transplant recipients represent a particularly vulnerable group, in whom chronic HEV infection can compromise both hepatic and graft outcomes (9).

Genotype 3 displays considerable genetic, geographic and host heterogeneity, with subgenotypes 3a-3f prevailing in Europe and North America and 3g-3j mainly reported in Asia (10, 11). Rabbit associated hepatitis E virus (raHEV) was first identified in farmed and wild rabbits in France between 2007 and 2010 (12), and subsequent molecular characterization established it as a distinct subgenotype within genotype 3 (HEV-3ra), widely distributed among rabbit populations across Europe and Asia (13). Recent surveillance studies have confirmed HEV-3ra or closely related clades in regions where rabbit farming and consumption are common (14). Experimental studies in immunocompromised rabbits have shown that both human and rabbit HEV strains can establish chronic infection, supporting potential for zoonotic transmission (15). Although a single report from Germany described chronic HEV infection in an immunosuppressed patient harboring a strain closely related to HEV-3ra (16), definite evidence of human infection has not yet been established.

Diagnosis of chronic hepatitis E in immunocompromised hosts is challenging because serology is often unreliable, requiring molecular confirmation (17). Management generally involves reduction of immunosuppressive therapy, which clears the virus in only a subset of patients, while ribavirin has become the mainstay of treatment in persistent cases (18–20). Although ribavirin is effective in treating chronic HEV infection, it can cause side effects, such as hemolytic anemia, requiring careful monitoring (21).

Herein, we report two long-term kidney transplant recipients who presented with elevated liver enzymes during routine follow-up and were subsequently diagnosed with chronic HEV genotype 3 infection, including the first documented case involving HEV-3ra, alongside a review of the literature documenting cases of chronic HEV infection in kidney transplant recipients

## Materials and methods

### Samples

Blood and stool samples were collected from both kidney transplant recipients for HEV molecular detection at diagnosis, at treatment initiation, and at 1-, 2-, 3-, and 6-months during therapy, as well as 3 and 6 months after ribavirin discontinuation. In parallel, blood analyses included complete blood count as well as liver and renal function tests.

### Sample preparation and RNA extraction for HEV detection

Blood samples were centrifuged at 3,000 rpm for 10 min for plasma separation and stool samples were pre-treated with InhibitEx buffer from QIAamp Fast DNA stool Mini Kit (Qiagen, Hilden, Germany), according to the manufacturer’s instructions. Nucleic acid was extracted from 400 μl of plasma and stool samples using the QIAmp DSP Virus Kit (Qiagen, Hilden, Germany) in the EZ1 Advanced (Qiagen) with a 60 μl elution volume.

### Real-time RT-PCR for HEV monitoring

For HEV monitoring extracts were screened individually using a broad-spectrum real-time RT-PCR (RT-qPCR) assay targeting the ORF3 region, using primers and a TaqMan probe as described previously (22). The RT-qPCR assays were performed using the iTaq Universal Probes One-Step Kit (Bio-Rad Laboratories, United States) with a final reaction volume of 20 μL, carried out on a CFX Connect Real-Time thermocycler (Bio-Rad Laboratories, United States). The thermal profile began with reverse transcription at 50 °C for 10 min, followed by reverse transcriptase inactivation and initial cDNA denaturation at 95 °C for 3 min. Amplification was then conducted over 45 cycles, each including denaturation at 95 °C for 15 s and annealing/extension at 55 °C for 15 s. The detection limit of the assay is 500 copies/mL.

### Nested RT-PCR for HEV genotyping

Hepatitis E virus genotyping was performed using a broad-spectrum nested RT-PCR assay targeting the RNA-dependent RNA-polymerase (*RdRp*) gene of the ORF1 region of the HEV genome (nt 331-334) using the outer primer set HEV-cs/HEV-cas (TCGCGCATCACMTTYTTCCARAA/GCCATGTTCCAGACDG TRTTCCA) and inner primer set HEV-csn/HEV-casn (TGTGCTCTGTTTGGCCCNTGGTTYCDG/CCAGGCTCACCR GARTGYTTCTTCC), spanning nt 4285–4616 (numbering according to genotype 3 strain Meng accession number AF082843), developed for detection of novel hepeviruses (23). For the first round, Qiagen One-Step RT-PCR kit (Qiagen^®^, Hilden, Germany) was used and for the second round, 5 μL of the first-round products were used as templates with GoTaq^®^ (Promega, WI, United States), all according to the manufacturer’s instructions. The WHO PEI 6329/10 subgenotype 3a standard (accession number AB630970, provided by the Paul Ehrlich-Institute, Langen, Germany) was used as a positive control and RNase-free water as negative control. Amplification reactions, with the corresponding positive and negative controls (nuclease – free water), were conducted in Bio-Rad T100TM Thermal Cycler. The conditions for the first round were an initial reverse transcription (RT) step for 15 min at 45 °C followed by 3 min at 95 °C (enzyme activation, denaturation of template DNA), 40 cycles of 95 °C for 15 s, 50 °C for 15 s, and 72 °C for 2 s, with a final elongation at 72 °C for 10 min. The second round followed the same conditions, excluding the RT step.

### Sequencing and phylogenetic analysis

Amplicons of the expected size that tested positive were purified using the GRS PCR & Gel Band Purification Kit (GriSP^®^). Following purification, bidirectional sequencing was performed with the Sanger method and appropriate internal specific primers for the target gene. The sequences were then aligned and compared to those in the NCBI (GenBank) nucleotide database, using the BioEdit Sequence Alignment Editor v7.1.9 software package, version 2.1. Phylogenetic analysis was performed using MEGA version X software (24). The maximum-likelihood (ML) approach was used to infer this analysis (24, 25), and General Time Reversible model was used to estimate the maximum likelihood (ML) bootstrap values using 1,000 replicates. The General Time Reversible was determined by MEGA version X as the best replacement (24). Further typing was performed with the HEVnet genotyping tool to identify HEV genotypes/subgenotypes (26).

## Results

The first patient was a 50-year-old male, 2 years post-kidney transplantation, who presented with elevated liver enzymes during routine follow-up. HEV infection was confirmed by RNA detection in blood, and chronic hepatitis E was assumed after persistent viremia despite reduction of immunosuppression. Ribavirin therapy was initiated, initially planned for 3 months, but extended to 6 months due to persistence of HEV RNA in stool after 2 months of therapy. Hemolysis during treatment required stepwise dose reductions, yet sustained virological clearance was achieved and maintained after therapy discontinuation.

The second patient was a 51-year-old male, 10 years post-transplantation, also diagnosed with HEV infection and chronic hepatitis E after unexplained elevations of aminotransferases and persistent viral replication after reduction of immunosuppression, respectively. Ribavirin was started at a dose adjusted according to renal function, later increased for tolerance, and was extended to 6 months after HEV RNA remained detectable in stool at month 2. Treatment was well-tolerated, and sustained clearance was confirmed after therapy.

The demographic and baseline clinical characteristics of the two kidney transplant recipients are summarized in Table 1, while Table 2 presents longitudinal laboratory follow-up findings, HEV RNA monitoring, and treatment outcomes.

**TABLE 1:** Demographic and clinical characteristics of hepatitis E virus (HEV)-3 infected kidney transplant patients.

Characteristics	Patient 1	Patient 2
Gender	Male	Male
Age (years old)	50	51
Year of transplant	2021	2013
Immunosuppressive regimen	Prednisolone 5 mg/day	Prednisolone 5 mg/day
	Mycophenolate mofetil 500 mg 12/12 h	Mycophenolate mofetil 750 mg 12/12 h
	Tacrolimus 11 mg/day	Tacrolimus 5 mg/day
Clinical presentation	Elevated liver enzymes	Elevated liver enzymes
	AST/ALT 90/57 U/L	AST/ALT 100/73 U/L
Onset of hepatitis	November 2023	October 2023
	HEV RNA+(blood)	HEV RNA+(blood)
Treatment adjustment	Tacrolimus reduction to 7.5 mg/day	Tacrolimus reduction to 4.5 mg/day
Evolution	December 2023	November 2023
	HEV RNA+(blood, stool)	HEV RNA+(blood, stool)
Hepatis A immunity	Yes	Yes
Hepatitis B carrier	No (vaccinated)	No (vaccinated)
Hepatitis C serology	Negative	Negative

AST, aspartate transaminase; ALT, alanine transaminase.

**TABLE 2:** Laboratory follow-up, hepatitis E virus (HEV) detection and treatment outcomes.

Characteristics	Patient 1	Patient 2
**Treatment start**	**December 2023 Ribavirin**	**December 2023 Ribavirin**
Initial dose	1,000 mg/day	200 mg/day (renal adjustment)
Dose adjustment	800 mg/day (hemolysis)	400 mg/day (tolerance)
Further adjustment	600 mg/day (hemolysis)	–
Month 0	HEV RNA+(blood, stool)	HEV RNA+(blood, stool)
Month 1	HEV RNA–(blood, stool)	HEV RNA–(blood, stool)
Month 2	HEV RNA+(stool only)	HEV RNA+(stool only)
Month 3	HEV RNA–(blood, stool)	HEV RNA–(blood, stool)
Month 6	HEV RNA–(blood, stool)	HEV RNA–(blood, stool)
Month 3 post-treatment	HEV RNA–(blood, stool)	HEV RNA–(blood, stool)
Month 6 post-treatment	HEV RNA–(blood, stool)	HEV RNA–(blood, stool)
Adverse events	Hemolysis (Hb nadir 9.7 g/dL)	None
Outcome	Sustained virological clearance	Sustained virological clearance

**FIGURE 1:**
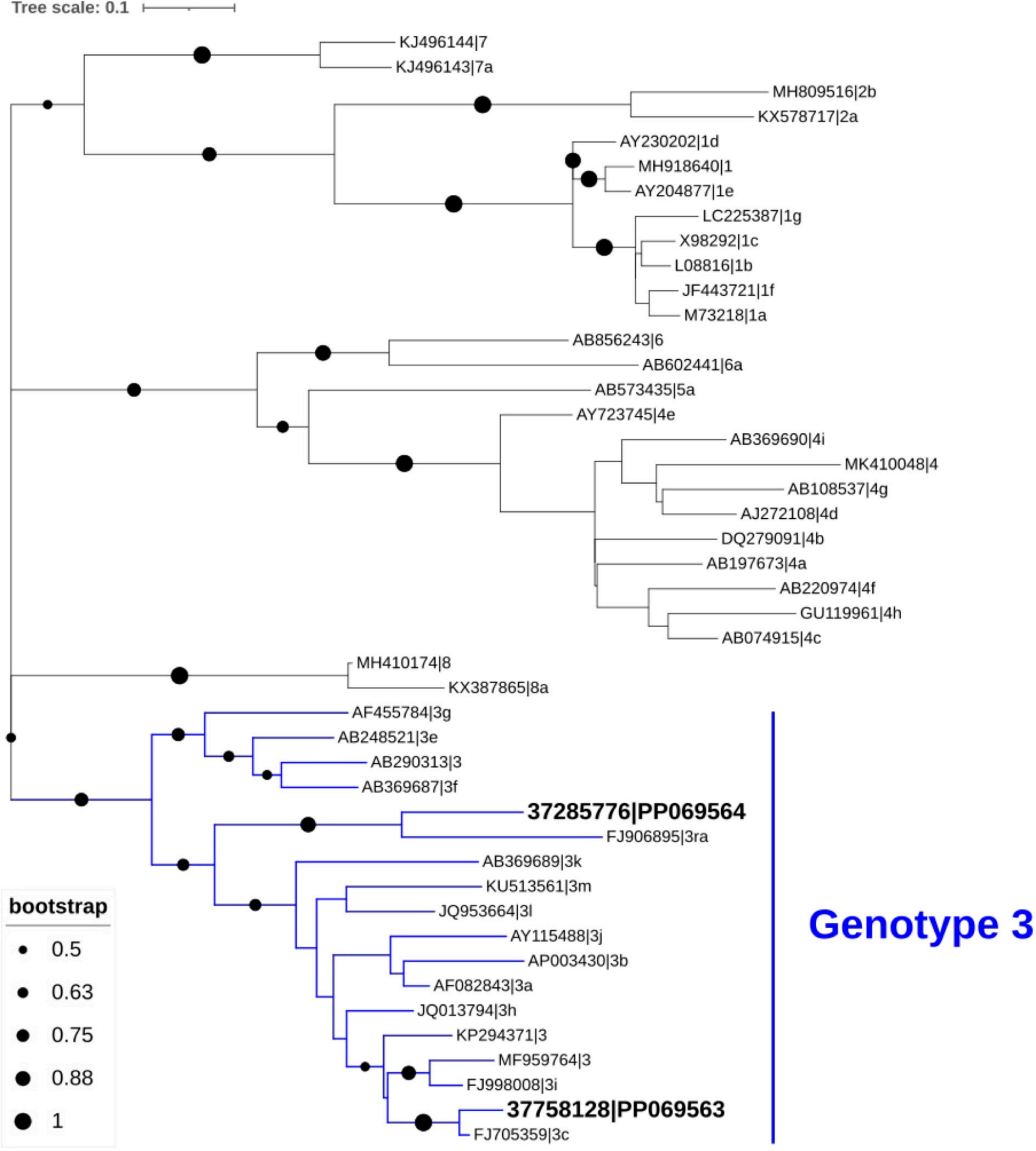
Phylogenetic analysis of hepatitis E virus (HEV) sequences identified in stool samples from the two patients. Genotypic clustering of these sequences (GenBank database accession number PP069563 and PP069564) confirmed their classification as subtypes 3c and 3ra. The phylogenetic tree was inferred using MEGA X and visualized with the Interactive Tree of Life (iTOL), based on 45 HEV nucleotide sequences, including 43 reference strains representing various genotypes obtained from GenBank.

Phylogenetic analysis of HEV sequences obtained from stool samples collected at month 2 from the two kidney transplant recipients revealed clustering within genotype HEV-3, specifically subgenotypes c and ra (Figure 1).

## Discussion

The present report describes two renal transplant recipients who developed chronic hepatitis E infection during post-transplant follow-up, occurring 2 and 10 years after transplantation, respectively. Both patients were diagnosed after investigation of abnormal liver function tests, in line with institutional protocol consistent with international clinical practice guidelines (27, 28). Initial reduction of immunosuppressive therapy was insufficient to achieve viral clearance, and ribavirin was required for 6 months. One patient required dose adjustment according to renal function, while the other developed hemolytic anemia after 1 month of therapy, which stabilized after ribavirin dose reduction. These cases illustrate the clinical challenges of balancing antiviral efficacy with hematologic toxicity in the renal transplant population.

To contextualize our findings and critically evaluate the appropriateness of our clinical approach, we conducted a review of the published literature on chronic HEV infection in kidney transplant recipients (Supplementary Table 1) (29–35). This analysis aimed to compare clinical presentations, therapeutic interventions, and outcomes across reported cases, thereby allowing assessment of concordance with existing evidence while also identifying areas where current knowledge remains limited. In this framework, our findings are largely consistent with prior reports. HEV infection in kidney transplant recipients is usually clinically silent, without jaundice, and associated with only modest ALT elevations, typically in the range of 100–300 IU/L. This pattern mirrors findings from prospective studies showing that nearly 60% of solid organ transplant recipients infected with HEV-3 progress to chronic infection (36). Consistent with previous data, both our patients received tacrolimus-based immunosuppression, which has been associated with a higher risk of viral persistence (37).

Natural history data further confirm the risk of rapid fibrosis as progressive liver disease is common, with approximately 10% of chronically HEV infected transplant recipients developing cirrhosis within 3–5 years, a rate faster than that observed in chronic HBV or HCV (36, 37). Early reports described progression to cirrhosis in less than 2 years (38), and more recent series have confirmed accelerated fibrinogenesis in this setting (39). Although such progression was not observed in our two patients during follow-up, these data highlight the potential severity of chronic HEV infection in renal transplant recipients and reinforce the need for systematic HEV surveillance to enable early diagnosis and timely treatment before advanced liver disease develops .

Therapeutic responses in our two cases also align with the published experience. Reduction of immunosuppression alone has been shown to achieve spontaneous clearance in about one-third of patients, particularly when T-cell-targeting agents are minimized (37, 40). However, the majority require ribavirin, which achieves sustained virological response in more than 80% of cases (21). Hematological toxicity is common, particularly hemolytic anemia, often necessitating dose modification (41), as occurred in Patient 1, yet virological clearance was ultimately achieved. Importantly, in our series ribavirin therapy was initiated only after approximately 1 month after diagnosis, once it became evident that immunosuppression reduction alone was insufficient to achieve viral clearance. This delay was also partly due to logistical barriers, including difficulties in the importation of ribavirin, which postponed timely initiation of treatment. Such real-world constraints highlight that treatment delays may contribute to viral persistence and should be considered when interpreting outcomes. Pegylated interferon has occasionally been used as salvage treatment but remains contraindicated in most transplant settings due to the risk of acute rejection (42).

Although chronic HEV infection is conventionally defined by the persistence of HEV RNA in blood or stool for at least 3 months (20), both patients in our series exhibited continued viremia after reduction of immunosuppression, indicating sustained viral replication. Because spontaneous viral clearance after this point is rarely observed in immunosuppressed individuals, ribavirin therapy was initiated without delay. Formal chronicity was subsequently confirmed during longitudinal follow-up according to the standard 3-month definition. This approach aligns with current clinical practice, where early persistence of viremia after immunosuppression reduction is considered a strong predictor of chronic evolution and warrants timely antiviral treatment to prevent progressive liver injury (36, 37).

The present study departs from existing literature in several noteworthy respects, particularly regarding the subgenotype detected and the inferred transmission route. To our knowledge, this is the first documented case of chronic HEV-3 subtype 3ra in a solid organ transplant recipient. Previous publications have described infection with HEV-3a, 3b, and 3c (39, 41), and sporadically with HEV-4 in East Asia (43–45). Other subtypes such as 3e, 3f, and rare recombinant variants like 3hi have been described in human and zoonotic reservoirs but not as a cause of chronic infection in kidney transplant recipients (11, 46, 47). Of particular relevance, subtype 3ra has been primarily reported in rabbits as a zoonotic reservoir, with phylogenetic analysis demonstrating close genetic clustering between rabbit-derived strains and human isolates (48). While this supports the pathogenic potential of HEV-3ra, especially under immunosuppression, documented cases in renal transplant recipients remains unconfirmed. However, given the close genetic relationship between HEV-3ra and human HEV-3, and the zoonotic nature of HEV, it represents a plausible risk — particularly in regions where rabbit meat consumption or exposure is common (49). While donor-derived HEV infections have been reported (50), the long interval since kidney transplantation makes this route unlikely in our patients, supporting autochthonous food-borne exposure as the most plausible source and consistent with predominant HEV-3 epidemiology in Europe.

Beyond rabbit HEV, increasing attention has been directed toward other zoonotic hepatotropic virus, particularly rat HEV (*Rocahepevirus ratti*). The first human infection was reported in Hong Kong in 2018, where rHEV presented with persistent hepatitis in a liver transplant recipient (51). Subsequent reports from Europe and North America have described severe acute hepatitis in both immunocompetent and immunocompromised individuals (52). Of particular concern, chronic rHEV infection can occur in in solid organ transplant recipients, mimicking the clinical and pathological profile of chronic HEV infection (53). Recognition of rHEV as a hepatotropic human pathogen expands the known diversity of the *Hepeviridae* family and underscores the need for molecular surveillance capable of distinguishing *Orthohepevirus A* (HEV genotypes 1–4) from potential emerging hepatotropic virus.

In summary, our findings not only reinforce the established features of chronic HEV infection in solid organ transplant recipients but also expand the current knowledge by documenting the first case of chronic HEV-3ra infection in a renal transplant recipient. The identification of 3ra not only broadens the spectrum of HEV variants known to persisting under immunosuppression but also underscores the zoonotic dimension of HEV transmission. Collectively, these findings emphasize the need for of systematic virological surveillance, judicious adjustment of immunosuppression, timely initiation of ribavirin therapy, and routine viral sequencing to guide clinical practice and epidemiological understanding in transplant medicine.

## Data Availability

The raw data supporting the conclusions of this article will be made available by the authors, without undue reservation.
