# Supplementary material for: Expanding the spectrum of chronic hepatitis E in kidney transplantation: first report of HEV-3ra infection and review of literature
Source: Front Med (Lausanne). 2025 Dec 8;12:1705331. doi: 10.3389/fmed.2025.1705331 (PMC12719084; doi:10.3389/fmed.2025.1705331)
Supplement: Supplementary file 1 [file Table_1.doc]

**Table S3.** Chronic HEV infection in kidney transplant recipients.

| **First author (Year)** | **Country** | **Study type** | **KTR patients with chronic HEV (%)** | **Immunosuppression** | **Genotype / Subtype** | **Transmission** | **Clinical presentation** | **Treatment** | **Dosing / duration** | **Outcome** | **Notes** | **Reference** |
| --- | --- | --- | --- | --- | --- | --- | --- | --- | --- | --- | --- | --- |
| Gérolami (2008) | France | Case report | 1 | NR | NR | Autochthonous (foodborne) | Rapidly progressive liver disease | NR | NR | Cirrhosis | First report of chronic HEV after kidney transplant | [23] |
| Meyrier (2012) | France | Case report | 1 | NR | NR | NR | Abnormal liver function tests | NR | NR | NR | Highlights underdiagnosis in KTR | [37] |
| Helleux (2012) | Belgium | Case report | 1 | NR | NR | NR | Anormal liver function tests | immunosuppression reduction | NR | NR | Highlights underdiagnosis in KTR | [38] |
| Moal (2013) | France | Cohort (single centre) | 12/16 (80%) | Various (tacrolimus common) | HEV-3 | Autochthonous (foodborne) | Abnormal liver function tests | immunosuppression reduction  (Ribavirin in non-clears) | NR | 54% cleared after IS reduction; 1 cirrhosis at 14 months | High chronicity in KTR | [24] |
| Breda (2014) | Portugal | Case series | 2 | NR | NR | NR | Abnormal liver function tests | NR | NR | NR | First report from Portugal | [39] |
| Bouts (2015) | Netherlands | Case series (pediatrics) | 2 | NR | NR | NR | Abnormal liver function tests | immunosuppression reduction | NR | Sustained virological response | Reports viral clearance without ribavirin | [40] |
| Wang (2017) | Switzerland | Case report | 1 | NR | HEV-3 (new strain near 3h) | NR | Abnormal liver function tests | NR | NR | NR | Full-length HEV-3 genome from KTR patients | [41] |
| Sridhar (2018) | Hong Kong | Cohort (single centre) | 3 | Prednisolone + Everolimus | HEV-4 | Autochthonous (foodborne) | Abnormal liver function tests | Ribavirin | 800 mg/day for 12 weeks | Sustained virologic response | Demonstrated HEV-4 chronicity in KTR patients | [29] |
| Wang (2018) | China | Case report | 1 | NR | HEV-4 | NR | Abnormal liver function tests | Ribavirin | NR | Sustained virologic response | First report of HEV-4 chronicity in KTR patients | [28] |
| Panning (2019) | Germany | Case report | 1 | Tacrolimus + MMF + Prednisolona; prior Rituximab | HEV-3c | Unknown (foodborne possible) | Abnormal liver function tests | Ribavirin | 200 mg x5/day for 11 weeks | Sustained virologic response; anemia led to early stop | Emphasizes molecular testing, due to reinfection despite antibodies | [26] |
| Van Wezel (2019) | Netherlands | Case series (mixed SOT) | 1 | NR | HEV-3 | NR | Chronic HEV refractory to ribavirin | Sofosbuvir + Ribavirin | NR | Sustained virologic response | Experimental salvage therapy after ribavirin failure | [42] |
| Owada (2020) | Japan | Cohort (nationwide) | 4/2526 | NR | HEV-3 | NR | HEV viremia | Ribavirin | 12 weeks | Sustained virologic response (100%) | HEV RNA prevalence in 11 patients; 4 patients with chronic HEV | [30] |
| Ollivier-Hourmand (2020) | France | Case report | 1 | NR | NR | NR | Chronic HEV refractory to ribavirin | Peg-IFN-alfa | NR | Sustained virologic response; no graft rejection | Experimental salvage therapy after ribavirin failure | [27] |
| Gruz (2020) | Argentina | Case report | 1 | NR | NR | NR | Abnormal liver function tests | Ribavirin | NR | Sustained virologic response | First Latin American KTR patient with chronic HEV treated with ribavirin | [43] |
| Solignac (2024) | France | Case report | 1 | NR | HEV-3c | Autochthonous (donor-derived) | Abnormal liver function tests | Ribavirin | NR | Sustained virologic response | First report of donor-derived HEV transmission | [36] |

KTR — kidney transplant recipients ; NR — Not referred
